# Machine Learning-Based Electroencephalographic Phenotypes of Schizophrenia and Major Depressive Disorder

**DOI:** 10.3389/fpsyt.2021.745458

**Published:** 2021-10-13

**Authors:** Kuk-In Jang, Sungkean Kim, Soo Young Kim, Chany Lee, Jeong-Ho Chae

**Affiliations:** ^1^Department of Cognitive Science Research, Korea Brain Research Institute (KBRI), Daegu, South Korea; ^2^Department of Human-Computer Interaction, Hanyang University, Ansan, South Korea; ^3^Department of Psychiatry, College of Medicine, The Catholic University of Korea, Seoul, South Korea

**Keywords:** electroencephalographic phenotyping, machine learning, auditory P300, schizophrenia, major depressive disorder

## Abstract

**Background:** Psychiatric diagnosis is formulated by symptomatic classification; disease-specific neurophysiological phenotyping could help with its fundamental treatment. Here, we investigated brain phenotyping in patients with schizophrenia (SZ) and major depressive disorder (MDD) by using electroencephalography (EEG) and conducted machine-learning-based classification of the two diseases by using EEG components.

**Materials and Methods:** We enrolled healthy controls (HCs) (*n* = 30) and patients with SZ (*n* = 34) and MDD (*n* = 33). An auditory P300 (AP300) task was performed, and the N1 and P3 components were extracted. Two-group classification was conducted using linear discriminant analysis (LDA) and support vector machine (SVM) classifiers. Positive and negative symptoms and depression and/or anxiety symptoms were evaluated.

**Results:** Considering both the results of statistical comparisons and machine learning-based classifications, patients and HCs showed significant differences in AP300, with SZ and MDD showing lower N1 and P3 than HCs. In the sum of amplitudes and cortical sources, the findings for LDA with classification accuracy (SZ vs. HCs: 71.31%, MDD vs. HCs: 74.55%), sensitivity (SZ vs. HCs: 77.67%, MDD vs. HCs: 79.00%), and specificity (SZ vs. HCs: 64.00%, MDD vs. HCs: 69.67%) supported these results. The SVM classifier showed reasonable scores between SZ and HCs and/or MDD and HCs. The comparison between SZ and MDD showed low classification accuracy (59.71%), sensitivity (65.08%), and specificity (54.83%).

**Conclusions:** Patients with SZ and MDD showed deficiencies in N1 and P3 components in the sum of amplitudes and cortical sources, indicating attentional dysfunction in both early and late sensory/cognitive gating input. The LDA and SVM classifiers in the AP300 are useful to distinguish patients with SZ and HCs and/or MDD and HCs.

## Introduction

Although psychiatric diagnosis is based on phenomenological distinction of overt features such as behavior, mood, and thought, determination of the neuropathological mechanisms with electroencephalography (EEG) remains challenging. Among the neurophysiologic phenotypes determined using EEG, there is no consistently recommended brain model for schizophrenia (SZ) and major depressive disorder (MDD). Previous EEG studies have focused on pathophysiological distinctions and symptomatic relationships (1–3). The clinical variations in SZ and MDD are very heterogeneous (4–6). Beyond clinical diagnosis based on phenomenological distinctions between SZ and MDD, the definition of EEG endophenotypes using machine learning could provide insights facilitating therapeutic breakthroughs for a variety of pathologic phenotypes (7–10). Especially, classification performance in psychiatric disorders was assured by applying linear discriminant analysis (LDA) and support vector machine (SVM) (11).

Auditory P300 (AP300) is a representative neurophysiological indicator in patients with SZ and depression (12–15); however, some studies provided inconsistent findings for this indicator in depression (16). AP300 includes the N1 and P3 components, which represent the most negative potential at around 100 ms and the most positive potential at around 300 ms following the onset of an auditory stimulus, respectively. Changes in P3 and N1 amplitudes in the midline electrodes are commonly observed (17, 18). Furthermore, the highest peak potential within defined time ranges for each component shows large variations across individuals because each component includes several neurobiological attributes (19, 20). Alternatively, the width of amplitudes within the defined time ranges can also indicate a pathological state (21).

AP300 reflects cognitive processes in auditory responses as well as working memory and attention process (22, 23). N1 has been defined as the neural allocation for early sensory input from the target stimulus (24, 25), and decreased N1 could reflect abnormal early selective attention in SZ and mood disorder (26–29). P3 is a major component of AP300 that is generated by late positive potential from information processing, such as an inputting rare event under ordinary situations (30, 31). N1 and P3 deficiencies are commonly observed in patients with SZ (32, 33). Several studies have also reported delayed latencies and decreased amplitudes of both N1 and P3 in patients with MDD (13, 34–36).

Here, we compared AP300 between healthy controls (HCs) and patients with SZ and MDD. To identify brain phenotypes of SZ and depression, changes in the N1 and P3 components were expressed in three dimensions, namely, peak with latency, sum of amplitudes, and cortical sources, by using radar charts. In addition, we applied machine learning techniques with linear discriminant analysis (LDA) and support vector machine (SVM) classifiers for each two-group classification.

## Materials and Methods

### Participants

We enrolled 34 patients with SZ (13 men and 21 women), 33 patients with MDD (11 men and 22 women), and 30 HCs (15 men and 15 women). The mean ages of the participants with SZ and MDD and the HCs were 37.21 ± 14.94, 40.03 ± 11.08, and 43.63 ± 12.80 years, respectively. The ages of all participants ranged from 19 to 82 years (mean: 40.15 ± 13.19 years). Participants who had vision or hearing problems, drug and/or alcohol abuse, traumatic brain injury, and a lifetime history of neurological disorders were excluded. Healthy participants with a lifetime history of psychiatric disorders were also excluded. All participants were native Koreans diagnosed using the MINI International Neuropsychiatric Interview of the *Diagnostic and Statistical Manual of Mental Disorders, 5th Edition*. The Positive and Negative Syndrome Scale (PANSS) (37) was evaluated in patients with SZ, while the Hamilton Depression and Anxiety rating scales (HAMD and HAMA) (38, 39) were evaluated in patients with MDD. The Beck Depression Inventory (BDI) was also evaluated in patients with MDD and HCs (40). All symptomatic evaluations were performed by a trained psychiatrist. Written informed consent was obtained from all the participants. This study followed the relevant guidelines and regulations of the Institutional Review Board of Seoul St. Mary's Hospital College of Medicine, The Catholic University of Korea (approval number: KC09FZZZ0211).

### EEG Measurements

All the participants were seated in a comfortable chair in a sound-attenuated room. The EEG recording was performed using the NeuroScan SynAmps amplifier (Compumedics USA, El Paso, TX, USA) with a 62-channel head cap mounted with AgCl electrodes according to the international extended 10–20 system (FP1, FPz, FP2, AF3, AF4, F7, F5, F3, F1, Fz, F2, F4, F6, F8, FT7, FC5, FC3, FC1, FCz, FC2, FC4, FC6, FT8, T7, C5, C3, C1, Cz, C2, C4, C6, T8, TP7, CP5, CP3, CP1, CPz, CP2, CP4, CP6, TP8, P7, P5, P3, P1, Pz, P2, P4, P6, P8, PO7, PO5, PO3, POz, PO4, PO6, PO8, CB1, O1, Oz, O2, and CB2). Eye movements were detected by electrooculography (EOG) sensors placed above and below the left eye and the outer canthus of both eyes. Bandpass filters ranged from 1 to 100 Hz with a sampling rate of 1000 Hz. The reference and ground channels were located on both the mastoids and forehead, respectively. The impedance was maintained below 5 kΩ during the recording session.

### AP300 Protocol and Analyses

AP300 with an auditory oddball task was conducted in the response-contingent behavior paradigm comprising 200 stimuli delivered using MDR-XB500 headphones (Sony, Tokyo, Japan) at 85 dB SPL with 2,000-ms fixed inter-stimulus intervals. A total of 160 standard tones of 1,000 Hz and 40 target tones of 1,500 Hz were presented randomly. The duration of the tone was 100 ms, and the rise and fall times were 10 ms. The STIM2 system (Compumedics USA, El Paso, TX, USA) was used to synchronize the auditory stimuli and EEG signals. All participants were instructed to press a button promptly when the target tones of 1,500 Hz were presented. A fixation cross was displayed in the middle of the screen during all recording sessions. Above 30-artifact free and -accurate epochs were used in the analyses. Gross artifacts were removed through visual inspection by a trained evaluator who had no information about the origin of the data. Artifacts related to eye blinks and/or movements were rejected in accordance with established mathematical procedures by using SCAN 4.5 and CURRY 8.0 software (41). Based on vertical EOG, positive and negative components exceeding 300 μV from the before- and after-onset stimuli (−100 to 300 ms) were rejected. The data were epoched from before-onset 100 ms to after-onset 700 ms on target stimuli. Pre-stimulus baseline correction was applied, and artifacts exceeding ±100 μV were rejected for all electrodes. The data were bandpass-filtered with a zero-phase shift ranging from 1 to 55 Hz. In the peak with latency, N1 was extracted between 50 and 150 ms post-stimulus. P3 was extracted between 250 and 500 ms. The width of the amplitudes was calculated by summation of all the amplitudes within the defined time ranges.

### Cortical Source Analyses and Regions of Interest (ROIs)

Cortical source estimation was performed using standardized low-resolution brain electromagnetic tomography (sLORETA) software. Estimation of the EEG inverse problem was conducted at the cortical source regions based on the 6,239 voxels (42). The source densities of N1 and P3 were calculated using mean values within the defined time ranges. ROIs in the cortical source level were selected to examine changes in the default mode network regions and cognitive control network (43, 44). The source activities of ROIs were extracted from the mean voxel values of the selected areas. The selected 14 regions were the left/right superior frontal gyri (SFGs), left/right middle frontal gyri (MFGs), left/right medial frontal gyri (MeFGs), left/right inferior frontal gyri (IFGs), left/right superior temporal gyri (STGs), left/right inferior parietal lobes (IPLs), and left/right precuneus.

### Machine Learning Analyses

Features were selected based on three dimensions: peak with latency (*n* = 12), sum of amplitudes (*n* = 7), and cortical sources (*n* = 28). Dimension-based feature selection was applied. The present study lacked a suitable sample size. Reducing dimension should be performed when the sample sizes and features were sufficiently large to secure acceptable classification performance (45). The classification accuracy, sensitivity, and specificity were evaluated using the 10-by-10-fold cross-validation technique with LDA (46) and linear SVM classifiers (47). Analysis in machine learning was conducted using MATLAB 2019 software with add on toolbox the Bioinformatics and the Statistics and Machine learning (Mathworks, Inc, USA).

### Statistical Analyses

Descriptive statistics were analyzed using multivariate analysis of variance (MANOVA), chi-square test, and *t*-test, as appropriate (Table 1). Age, education, and accepted AP300 trials among the groups were compared using MANOVA. Differences in sex were also examined using the chi-square test. BDI scores between patients with MDD and HCs were compared using *t*-test. For multivariate analysis with covariance, 49 variables of AP300 were examined as dependent variables among all groups, with age, sex, and education as covariates. Statistical significance was set at *p* < 0.05, two-tailed. Main-effect comparison was performed using the Bonferroni correction from the original *p*-values (48). All statistical analyses were performed using IBM SPSS software (version 20.0; IBM Corp., Armonk, NY, USA).

**Table 1:** Demographic data of the present study.

**Variables**	**SZ (*n* = 34) (a)**	**MDD (*n* = 33) (b)**	**HCs (*n* = 30) (c)**	**Statistics**
Age	37.21 (14.94)	40.03 (11.08)	43.63 (12.80)	f = 1.930, *p* = 0.151
Sex (m/f)	13/21	11/22	15/15	χ^2^, *p* = 0.387
Education	13.21 (3.37)	13.70 (2.30)	15.27 (1.57)	f = 5.553, *p* = 0.008 a < c
Duration of illness (Missing value)	29.09 (12.11) (0)	37.29 (7.83) (26)	-	-
Positive	29.26 (6.23)	-	-	-
Negative	19.97 (7.14)	-	-	-
General	52.94 (8.66)	-	-	-
Total	102.18 (15.08)	-	-	-
HAM-D	-	20.15 (5.65)	-	-
HAM-A	-	22.48 (7.77)	-	-
BDI	-	28.06 (12.33)	9.33 (7.49)	T = 7.357, *p* <0.001
AP300 accepted trials	37.71 (2.51)	37.27 (2.83)	37.73 (2.78)	F = 0.297, *p* = 0.744
Drug administration (*n*)	29	6	-	-
**Antipsychotics**				
Amisulpride	6	-	-	-
Aripiprazole	4	-	-	-
Blonanserin	1	-	-	-
Clozapine	1	-	-	-
Olanzapine	11	-	-	-
Paliperidone	5	-	-	-
Quetiapine	7	-	-	-
Risperidone	1	-	-	-
**Antidepressants**				
Alprazolam	-	1	-	-
Lorazepam	-	2	-	-
Mirtazapine	-	1	-	-
Paroxetine	-	1	-	-
Sertraline	-	1	-	-
Venlafaxine	-	3	-	-

## Results

Descriptive statistics are presented in Table 1. We found no significant differences in age (F = 1.930, *p* = 0.151) and sex (χ^2^, *p* = 0.387) among the three groups. Level of education was significant between patients with SZ and HCs (SZ < HCs, F = 5.553, *p* = 0.008). The BDI scores significantly differed between HCs and patients with MDD (t = 7.357, *p* < 0.001). The number of accepted AP300 trials did not differ significantly among the three groups (F = 0.297, *p* = 0.744).

**Figure 1:**
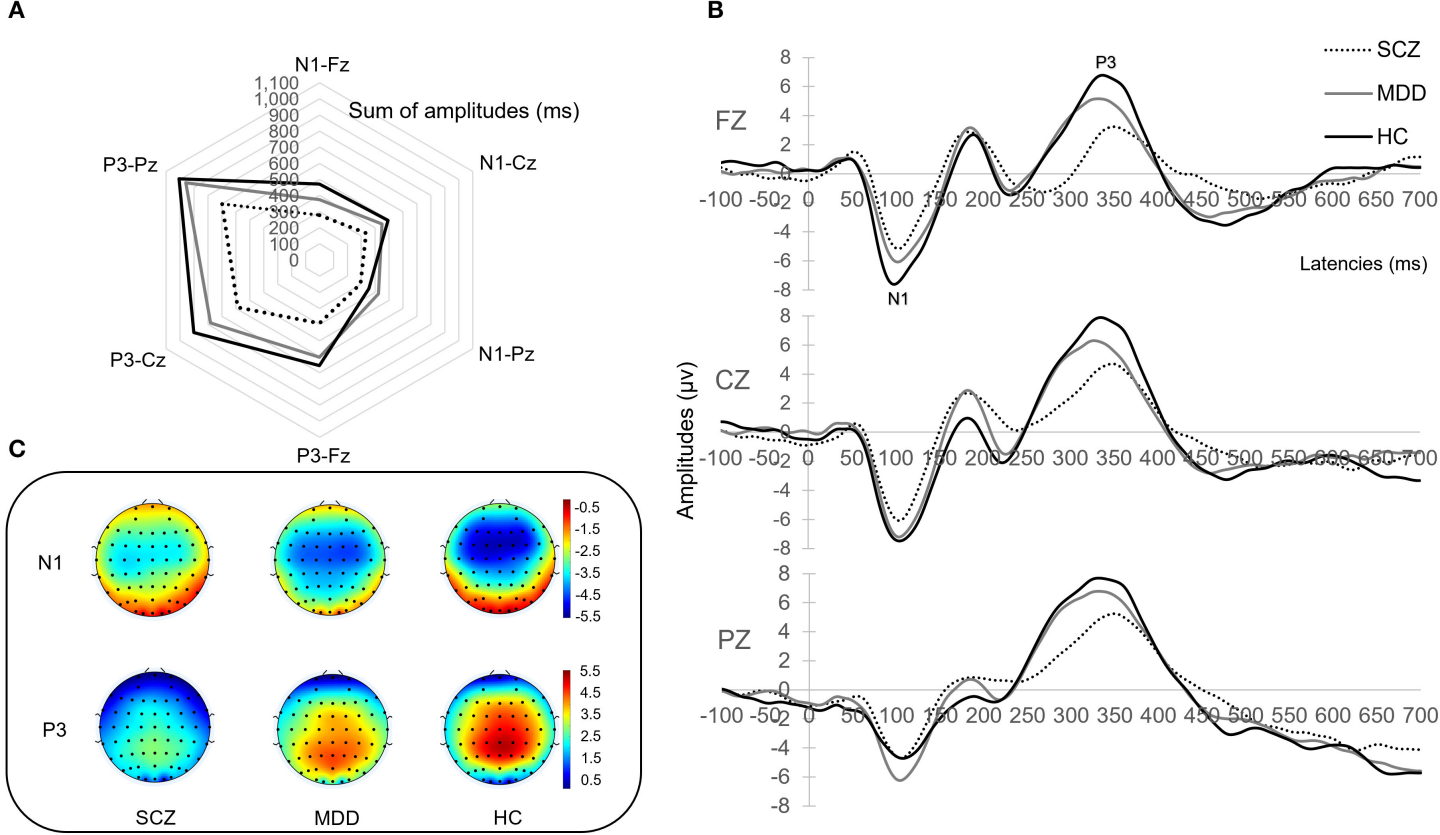
AP300 with brain phenotyping. **(A)** is the radar chart with sum of amplitudes. **(B)** is the peak and latency. **(C)** illustrates topographical map of N1 and P3 between patients and healthy controls.

The present study found significant differences in AP300 among HCs and patients with SZ and MDD [*F*_(2, 91)_ = 1.704, *p* = 0.006, η^2^ = 0.645]. In the assessment of the sum of amplitudes of AP300 (Table 2 and Figure 1A), significant differences were found among patients with SZ, MDD, and HCs (SZ < MDD < HCs, N1-Fz, *p* < 0.001; SZ < MDD, SZ < HCs, N1-Cz, *p* = 0.005; SZ < MDD, N1-Pz, *p* < 0.037; SZ < MDD, SZ < HC, Total value, *p* = 0.014).

**Table 2:** Comparisons of AP300 between patients and HCs.

**Variables**	**SZ (*n* = 34)**	**MDD (*n* = 33)**	**HCs (*n* = 30)**	**Statistics**			
	**(a)**	**(b)**	**(c)**	**Pairwise comparison**	**Original *p*-value**	**Bonferroni corrected *p*-value**	**Effect size (η^2^)**
**Sum of amplitudes**							
N1-Fz	278.18 (131.35)	375.58 (137.70)	471.05 (159.07)	a < b < c	<0.001	<0.001	0.226
N1-Cz	334.39 (117.94)	449.97 (162.03)	490.52 (144.58)	a < b, a < c	<0.001	0.005	0.182
N1-Pz	294.24 (121.56)	421.81 (164.11)	354.74 (102.65)	a < b	0.001	0.037	0.146
Total	2583.68 (1114.06)	3591.49 (1583.27)	3879.94 (1245.18)	a < b, a < c	<0.001	0.014	0.165
**Peak(μV) with latency (ms)**							
N1-Fz-Peak	6.00 (2.50)	7.37 (2.26)	8.46 (2.65)	a < c	0.001	0.027	0.152
P3-Pz-Peak	6.88 (3.33)	8.87 (3.72)	9.95 (3.52)	a < b, a < c	0.001	0.043	0.143
**Cortical sources**							
N1-Right IPL	0.41 (0.38)	0.39 (0.42)	0.82 (0.67)	a < c, b < c	<0.001	0.012	0.168
N1-Left Precuneus	0.89 (0.59)	0.91 (0.96)	1.67 (1.20)	a < c, b < c	<0.001	0.004	0.187
N1-Right Precuneus	0.80 (0.51)	0.88 (0.72)	1.51 (0.97)	a < c, b < c	<0.001	<0.001	0.223
P3-Right STG	0.86(0.52)	1.29 (0.87)	1.73 (0.88)	a < c, b < c	<0.001	0.003	0.194
P3-Left IPL	0.45(0.43)	0.50 (0.45)	1.12 (0.90)	a < c, b < c	<0.001	<0.001	0.245
P3-Right IPL	0.38(0.28)	0.35 (0.31)	0.97 (0.77)	a < c, b < c	<0.001	<0.001	0.250
P3-Left Precuneus	0.91(0.65)	1.14 (1.08)	2.66 (2.12)	a < c, b < c	<0.001	<0.001	0.276
P3-Right Precuneus	0.90(0.58)	1.09 (0.98)	2.38 (1.94)	a < c, b < c	<0.001	<0.001	0.244

**Figure 2:**
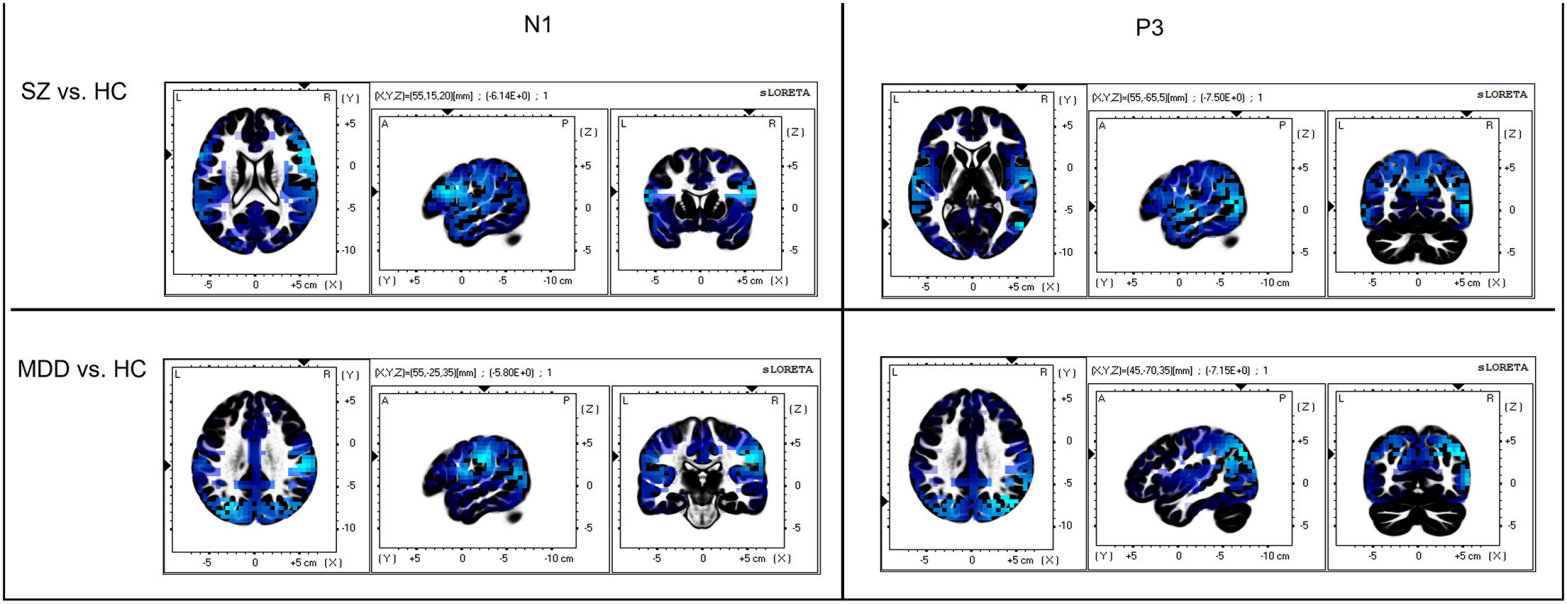
Comparison of AP300 at cortical source level. Blue color indicates cortical source deactivations in patients, compared to healthy controls. The sLORETA has a low-resolution anatomical distribution.

**Figure 3:**
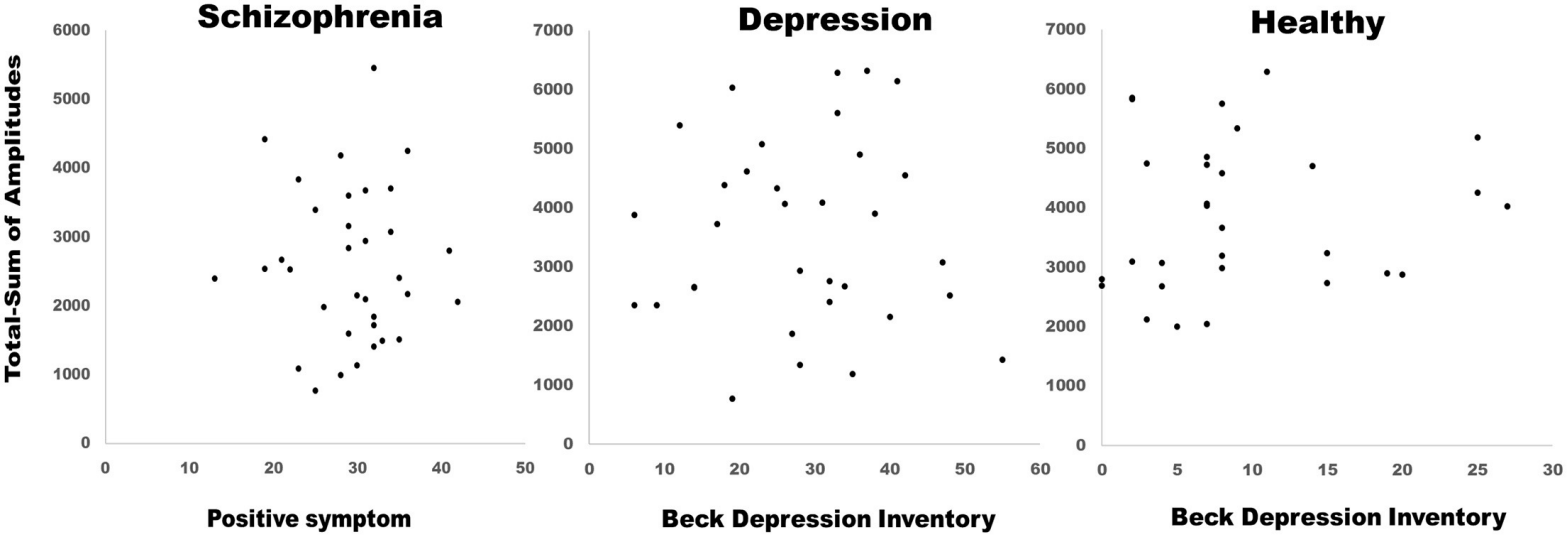
Correlations between clinical symptoms and AP300. Correlations were not significant.

On evaluating the peak with latency of AP300 (Table 2 and Figures 1B,C), differences were significant (SZ < HCs, N1-Fz-Peak, *p* = 0.027; SZ < MDD, SZ < HCs, P3-Pz-Peak, *p* = 0.043). On evaluating the cortical source activities of AP300 (Table 2 and Figure 2), significant differences were found (SZ < HCs, MDD < HCs, N1-R-IPL, *p* = 0.012; N1-L-Precuneus, *p* = 0.004; N1-R- Precuneus, *p* < 0.001; P3-R-STG, *p* = 0.003; P3-L-IPL, *p* < 0.001; P3-R-IPL, *p* < 0.001; P3-L-Precuneus, *p* < 0.001; P3-R-Precuneus, *p* < 0.001). There were no significant differences in the assessments of AP300 behavior. Meanwhile, correlations between clinical symptoms and AP300 were not significant (Figure 3).

The machine learning results with LDA and SVM are presented in Table 3. The results of the two-group classification are as follows:

**Accuracy for sum of amplitudes:** SZ vs. MDD, LDA, 59.71%, SVM, 54.48%; SZ vs. HCs, LDA, 71.31%, SVM, 57.81%; and MDD vs. HCs, LDA, 74.55%, SVM, 58.89%. Sensitivity for sum of amplitudes: SZ vs. MDD, LDA, 65.08%, SVM, 56.18%; SZ vs. HCs, LDA, 77.67%; SVM, 56.77%; and MDD vs. HCs, LDA, 79.00%, SVM, 57.58%. Specificity for sum of amplitudes: SZ vs. MDD, LDA, 54.83%, SVM, 52.73%; SZ vs. HCs, LDA, 64.00%, SVM, 59.00%; and MDD vs. HCs, LDA, 69.67%, SVM, 60.33%.
**Accuracy for peak with latency:** SZ vs. MDD, LDA, 55.75%, SVM, 53.88%; SZ vs. HCs, LDA, 70.74%, SVM, 67.35%; and MDD vs. HCs, LDA, 70.60%, SVM, 70.95%. Sensitivity for peak with latency: SZ vs. MDD, LDA, 57.25%, SVM, 55.00%; SZ vs. HCs, LDA, 69.42%; SVM, 70.00%; and MDD vs. HCs, LDA, 75.17%, SVM, 72.12%. Specificity for peak with latency: SZ vs. MDD, LDA, 55.00%, SVM, 52.73%; SZ vs. HCs, LDA, 72.33%; SVM, 64.33%; and MDD vs. HCs, LDA, 65.33%, SVM, 69.67%.
**Accuracy for cortical sources:** SZ vs. MDD, LDA, 54.28%, SVM, 54.78%; SZ vs. HCs, LDA, 65.41%, SVM, 71.88%; and MDD vs. HCs, LDA, 55.50%, SVM, 65.87%. Sensitivity for cortical sources: SZ vs. MDD, LDA, 58.25%, SVM, 66.77%; SZ vs. HCs, LDA, 69.25%, SVM, 74.41%; and MDD vs. HCs, LDA, 64.00%, SVM, 81.21%. Specificity for cortical sources: SZ vs. MDD, LDA, 50.08%, SVM, 42.42%; SZ vs. HCs, LDA, 60.67%, SVM, 69.00%; and MDD vs. HCs, LDA, 46.67%, SVM, 49.00%.

**Table 3:** Classification using LDA and SVM.

**Two sample classifications**	**Accuracy (%)**		**Sensitivity (%)**		**Specificity (%)**		**Number of features**	**Selected features**
	**LDA**	**SVM**	**LDA**	**SVM**	**LDA**	**SVM**		
SZ vs. MDD	59.71	54.48	65.08	56.18	54.83	52.73	7	Sum of amplitudes
SZ vs. HCs	71.31	57.81	77.67	56.77	64.00	59.00		
MDD vs. HCs	74.55	58.89	79.00	57.58	69.67	60.33		
SZ vs. MDD	55.75	53.88	57.25	55.00	55.00	52.73	12	Peak with latency
SZ vs. HCs	70.74	67.35	69.42	70.00	72.33	64.33		
MDD vs. HCs	70.60	70.95	75.17	72.12	65.33	69.67		
SZ vs. MDD	54.28	54.78	58.25	66.77	50.08	42.42	28	Cortical sources
SZ vs. HCs	65.41	71.88	69.25	74.41	60.67	69.00		
MDD vs. HCs	55.50	65.87	64.00	81.21	46.67	49.00		

## Discussion

The present study demonstrated differences in AP300 between HCs and patients with SZ and MDD. AP300 with N1 deficiency in patients with SZ and MDD was predominantly found in the sum of the amplitudes. Machine learning-based classification with LDA showed reasonable accuracy and sensitivity between SZ and HCs and/or MDD and HCs. Considering the results of both statistical comparisons and machine learning-based classification, patients with SZ showed defective EEG phenotypes in N1-Fz, N1-Cz, N1-Pz, and total value in the sum of amplitudes. Patients with MDD showed an impaired EEG phenotype in N1-Fz in the sum of amplitudes. In cortical sources, patients with SZ and MDD showed decreased N1 and P3. The SVM classifier showed reasonable sensitivity between SZ and HCs and/or MDD and HCs.

The impaired N1 component in patients with SZ reflects early sensory gating deficits, which lead to a dysfunctional process of attentional information (49). This impaired phenotype is associated with aberrant neural plasticity in SZ patients showing clinical high-risk factors (50). Patients with depression showed delayed latency of N1 and a lower P3 amplitude (36). Deficits in early sensory gating are related to maladaptive initial directions of sensory information, resulting in delayed N1 latency and lower amplitude (49). P3 is an index of the late sensory gating that decodes whether the stimulus is significant or unnecessary (51).

The present study showed significant differences in N1 and P3 between patients with SZ and those with MDD. Compared to patients with MDD, patients with SZ had lower N1 and P3 amplitudes. However, this difference lacked power because classification with machine learning has low accuracy, sensitivity, and specificity. Previous studies reported that high classification performance was identified when sensor and source level EEG features were used together (10). EEG microstate features had higher classification performance than conventional EEG features in patients with SZ (52). In MDD, EEG band frequency features showed a good performance classifying patients and healthy individuals (53). In the present study, the mean and standard deviation in the EEG data could influence the results in statistical comparison, while distributional similarity of the used features between groups could have a possible effect on the lacking power in classification with machine learning. In addition, sociodemographic factors could influence the results. Further studies are warranted in patients with several clinical phenotypes and EEG features.

This study had a few limitations. First, the sample size was small; thus, future studies with large sample sizes should be conducted to verify the results. Second, several clinical phenotypes, such as affective or mood-specific types and psychosis with mood symptoms, need to be considered. Nevertheless, determination of the neuropathological mechanism via EEG phenotyping could provide useful information for the fundamental treatment of psychiatric disorders. This study identified that in sum of amplitude, a neurophysiologic phenotype with an N1 deficit featured in patients with MDD and SZ, indicating a dysfunctional process of early sensory attentional information. Supporting this result was that the LDA classifier showed reasonable accuracy and sensitivity. In cortical sources, a phenotype with deficits in both N1 and P3 was observed in patients with MDD and SZ, reflecting maladaptive early and/or late sensory/cognitive gating inputs. The SVM classifier with sensitivity showed reasonable scores.

## Data Availability Statement

The data presented in the current study are available from the first author (K-IJ) or corresponding author (J-HC) upon reasonable request. Requests to access these datasets should be directed to K-IJ.

## Ethics Statement

The studies involving human participants were reviewed and approved by Institutional Review Board of Seoul St. Mary's Hospital College of Medicine, The Catholic University of Korea (approval number: KC09FZZZ0211). The patients/participants provided their written informed consent to participate in this study.

## Author Contributions

K-IJ, CL, and J-HC contributed to the conception and design of the study. K-IJ, SYK, and CL contributed to the acquisition and analysis of data. K-IJ contributed to drafting the article. K-IJ, SK, CL, SYK, and J-HC contributed to the review of the article. J-HC and CL contributed to the supervision of the study. All authors approved the final version of the article.

## Funding

This study was supported by a grant from the Korea Health Technology R&D project through the Korea Health Industry Development Institute (KHIDI) (HI17C2272), as well as by the KBRI basic research program through the Korea Brain Research Institute, which is funded by the Ministry of Science and ICT (21-BR-01-13). This study was also supported by Basic Science Research Program through the National Research Foundation of Korea (NRF) funded by the Ministry of Education (2021R1A6A3A01086699).

## Conflict of Interest

The authors declare that the research was conducted in the absence of any commercial or financial relationships that could be construed as a potential conflict of interest.

## Publisher's Note

All claims expressed in this article are solely those of the authors and do not necessarily represent those of their affiliated organizations, or those of the publisher, the editors and the reviewers. Any product that may be evaluated in this article, or claim that may be made by its manufacturer, is not guaranteed or endorsed by the publisher.
