# Supplementary material for: Machine Learning-Based Electroencephalographic Phenotypes of Schizophrenia and Major Depressive Disorder
Source: Front Psychiatry. 2021 Oct 13;12:745458. doi: 10.3389/fpsyt.2021.745458 (PMC8549692; doi:10.3389/fpsyt.2021.745458)
Supplement: Supplementary file 1 [file Table_1.docx]

Supplementary Table. Comparisons in all used AP300 features between patients and HCs.

| **Variables** | **SZ(n=34)**  **(a)** | **MDD(n=33)**  **(b)** | **HCs(n=30)**  **(c)** | **Statistics** | | | |  |
| --- | --- | --- | --- | --- | --- | --- | --- | --- |
| ***Sum of Amplitudes*** |  |  |  | Pairwise  comparison | Original  p-value | Bonferroni corrected  p-value | Effect size  (η^2^) |  |
| N1-Fz | 278.18(131.35) | 375.58(137.70) | 471.05(159.07) | a<b<c | <0.001 | **<0.001** | 0.226 |  |
| N1-Cz | 334.39(117.94) | 449.97(162.03) | 490.52(144.58) | a<b, a<c | <0.001 | **0.005** | 0.182 |  |
| N1-Pz | 294.24(121.56) | 421.81(164.11) | 354.74(102.65) | a<b | 0.001 | **0.037** | 0.146 |  |
| P3-Fz | 392.25(275.55) | 603.50(390.88) | 654.71(373.37) |  | 0.007 | 0.346 | 0.103 |  |
| P3-Cz | 586.15(358.91) | 781.20(459.63) | 900.99(404.21) |  | 0.008 | 0.374 | 0.102 |  |
| P3-Pz | 698.48(362.33) | 959.43(460.81) | 1007.93(354.82) |  | 0.001 | 0.073 | 0.133 |  |
| Total | 2583.68(1114.06) | 3591.49(1583.27) | 3879.94(1245.18) | a<b, a<c | <0.001 | **0.014** | 0.165 |  |
| ***Peak*(μV)*with latency*(ms)** |  | | | | | | |  |
| N1-Fz-Peak | 6.00(2.50) | 7.37(2.26) | 8.46(2.65) | a<c | 0.001 | **0.027** | 0.152 |  |
| N1-Fz-Latency | 104.41(13.02) | 107.76(20.17) | 104.87(15.55) |  | 0.437 | 1.000 | 0.018 |  |
| N1-Cz-Peak | 6.78(2.27) | 8.46(2.67) | 8.64(2.20) |  | 0.002 | 0.109 | 0.126 |  |
| N1-Cz-Latency | 104.18(10.78) | 107.03(16.19) | 105.63(14.65) |  | 0.459 | 1.000 | 0.017 |  |
| N1-Pz-Peak | 5.58(1.87) | 7.29(2.69) | 6.00(1.38) |  | 0.005 | 0.236 | 0.111 |  |
| N1-Pz-Latency | 106.97(13.12) | 106.18(14.25) | 106.37(20.65) |  | 0.976 | 1.000 | 0.001 |  |
| P3-Fz-Peak | 5.49(3.28) | 7.83(3.83) | 8.34(3.74) |  | 0.002 | 0.108 | 0.126 |  |
| P3-Fz-Latency | 365.97(51.40) | 337.79(43.65) | 343.97(18.02) |  | 0.008 | 0.413 | 0.100 |  |
| P3-Cz-Peak | 6.71(3.51) | 8.93(4.08) | 9.93(3.85) |  | 0.001 | 0.068 | 0.135 |  |
| P3-Cz-Latency | 345.41(36.24) | 336.55(42.39) | 334.30(27.39) |  | 0.273 | 1.000 | 0.028 |  |
| P3-Pz-Peak | 6.88(3.33) | 8.87(3.72) | 9.95(3.52) | a<b, a<c | 0.001 | **0.043** | 0.143 |  |
| P3-Pz-Latency | 351.85(35.70) | 348.30(34.49) | 332.63(28.05) |  | 0.063 | 1.000 | 0.059 |  |
| ***Cortical sources*** |  | | | | | | |  |
| N1-Left SFG | 1.35(1.18) | 1.26(0.62) | 1.37(0.78) |  | 0.795 | 1.000 | 0.005 |  |
| N1-Right SFG | 1.43(1.37) | 1.37(0.78) | 1.88(1.81) |  | 0.168 | 1.000 | 0.038 |  |
| N1-Left MFG | 0.86(0.75) | 0.90(0.41) | 0.90(0.35) |  | 0.962 | 1.000 | 0.001 |  |
| N1-Right MFG | 0.96(0.78) | 1.14(0.69) | 1.38(1.24) |  | 0.180 | 1.000 | 0.037 |  |
| N1-Light MeFG | 1.28(0.99) | 1.24(0.58) | 1.43(0.98) |  | 0.426 | 1.000 | 0.019 |  |
| N1-Right MeFG | 1.27(1.04) | 1.24(0.58) | 1.60(1.35) |  | 0.149 | 1.000 | 0.041 |  |
| N1-Left IFG | 1.02(0.95) | 1.20(0.61) | 1.23(0.63) |  | 0.591 | 1.000 | 0.011 |  |
| N1-Right IFG | 1.15(0.95) | 1.44(0.94) | 1.74(1.32) |  | 0.108 | 1.000 | 0.048 |  |
| N1-Left STG | 0.86(0.67) | 1.08(0.58) | 1.41(0.57) |  | 0.009 | 0.420 | 0.099 |  |
| N1-Right STG | 0.98(0.69) | 1.22(0.72) | 1.61(0.99) |  | 0.020 | 0.964 | 0.083 |  |
| N1-Left IPL | 0.48(0.39) | 0.51(0.54) | 0.86(0.69) |  | 0.002 | 0.111 | 0.125 |  |
| N1-Right IPL | 0.41(0.38) | 0.39(0.42) | 0.82(0.67) | a<c, b<c | <0.001 | **0.012** | 0.168 |  |
| N1-Left Precuneus | 0.89(0.59) | 0.91(0.96) | 1.67(1.20) | a<c, b<c | <0.001 | **0.004** | 0.187 |  |
| N1-Right Precuneus | 0.80(0.51) | 0.88(0.72) | 1.51(0.97) | a<c, b<c | <0.001 | **<0.001** | 0.223 |  |
| P3-Left SFG | 1.97(2.08) | 1.87(0.93) | 2.39(1.26) |  | 0.100 | 1.000 | 0.049 |  |
| P3-Right SFG | 2.20(2.32) | 1.95(1.11) | 2.50(1.24) |  | 0.297 | 1.000 | 0.026 |  |
| P3-Left MFG | 1.16(1.08) | 1.29(0.67) | 1.54(0.64) |  | 0.057 | 1.000 | 0.061 |  |
| P3-Right MFG | 1.32(1.51) | 1.36(0.87) | 1.59(0.64) |  | 0.579 | 1.000 | 0.012 |  |
| P3-Left MeFG | 2.00(1.78) | 2.06(1.07) | 2.56(1.38) |  | 0.040 | 1.000 | 0.068 |  |
| P3-Right MeFG | 2.02(1.71) | 2.05(1.09) | 2.49(1.24) |  | 0.094 | 1.000 | 0.051 |  |
| P3-Left IFG | 1.33(1.15) | 1.77(1.05) | 2.02(1.23) |  | 0.032 | 1.000 | 0.073 |  |
| P3-Right IFG | 1.20(0.81) | 1.78(1.26) | 1.96(0.96) |  | 0.008 | 0.405 | 0.100 |  |
| P3-Left STG | 0.89(0.68) | 1.27(0.71) | 1.57(0.78) |  | 0.001 | 0.072 | 0.134 |  |
| P3-Right STG | 0.86(0.52) | 1.29(0.87) | 1.73(0.88) | a<c, b<c | <0.001 | **0.003** | 0.194 |  |
| P3-Left IPL | 0.45(0.43) | 0.50(0.45) | 1.12(0.90) | a<c, b<c | <0.001 | **<0.001** | 0.245 |  |
| P3-Right IPL | 0.38(0.28) | 0.35(0.31) | 0.97(0.77) | a<c, b<c | <0.001 | **<0.001** | 0.250 |  |
| P3-Left Precuneus | 0.91(0.65) | 1.14(1.08) | 2.66(2.12) | a<c, b<c | <0.001 | **<0.001** | 0.276 |  |
| P3-Right Precuneus | 0.90(0.58) | 1.09(0.98) | 2.38(1.94) | a<c, b<c | <0.001 | **<0.001** | 0.244 |  |
| ***AP300 behavior*** |  |  |  |  | | | |  |
| Accuracy | 38.41(2.32) | 39.24(1.091) | 39.37(1.30) |  | 0.108 | 1.000 | 0.048 |  |
| Reaction time (seconds) | 0.49(0.15) | 0.41(0.13) | 0.37(0.09) |  | 0.003 | 0.164 | 0.118 |  |
